# A Self-Powered Insole for Human Motion Recognition

**DOI:** 10.3390/s16091502

**Published:** 2016-09-15

**Authors:** Yingzhou Han, Yalu Cao, Jingjing Zhao, Yajiang Yin, Liangchen Ye, Xiaofeng Wang, Zheng You

**Affiliations:** 1 Collaborative Innovation Center for Micro/Nano Fabrication, Device and System, Tsinghua University, Beijing 100084, China; hanyz15@mails.tsinghua.edu.cn (Y.H.); caoyl15@mails.tsinghua.edu.cn (Y.C.); jing.jing.youxiang@163.com (J.Z.); yin-yj14@mails.tsinghua.edu.cn (Y.Y.); ylc12@mails.tsinghua.edu.cn (L.Y.); 2 State Key Laboratory of Precision Measurement Technology and Instruments, Tsinghua University, Beijing 100084, China; 3 Department of Precision Instrument, Tsinghua University, Beijing 100084, China

**Keywords:** wearable sensors, motion recognition, self-powered insole, energy harvester

## Abstract

Biomechanical energy harvesting is a feasible solution for powering wearable sensors by directly driving electronics or acting as wearable self-powered sensors. A wearable insole that not only can harvest energy from foot pressure during walking but also can serve as a self-powered human motion recognition sensor is reported. The insole is designed as a sandwich structure consisting of two wavy silica gel film separated by a flexible piezoelectric foil stave, which has higher performance compared with conventional piezoelectric harvesters with cantilever structure. The energy harvesting insole is capable of driving some common electronics by scavenging energy from human walking. Moreover, it can be used to recognize human motion as the waveforms it generates change when people are in different locomotion modes. It is demonstrated that different types of human motion such as walking and running are clearly classified by the insole without any external power source. This work not only expands the applications of piezoelectric energy harvesters for wearable power supplies and self-powered sensors, but also provides possible approaches for wearable self-powered human motion monitoring that is of great importance in many fields such as rehabilitation and sports science.

## 1. Introduction

With the rapid development of wearable electronics, there is an increasing demand for wearable and portable power sources. Traditional power sources such as batteries are relatively large and heavy comparing with some wearable sensors and need to be replaced or charged periodically, which makes them unfavorable for wearable power sources. Biomechanical energy harvesting is a promising wearable power source that has received intensive attention. First, it has the potential to sustainably drive wearable electronics, which could conceptually endow electronics with unlimited lifetimes. Second, a biomechanical energy harvester itself can serve as a wearable self-powered sensor which can accurately detect both static and dynamic processes of environmental triggers without external power sources. Last but not least, biomechanical energy harvesting is an environmentally-friendly and economically feasible technique, which is of great interest with the increasing public concerns about the energy crisis and climate change. A large number of biomechanical energy harvesters based on various mechanisms have been proposed, such as electromagnetic generators [1,2,3,4], electrostatic generators [5,6], thermoelectric generators [7,8,9,10], triboelectric nanogenerators [11,12,13,14] and piezoelectric generators [15,16,17,18,19,20]. Among these energy harvesters, electromagnetic generators have cumbersome structures and small output voltage at low frequencies [3]; the electrostatic and thermoelectric generators have low outputs during biomechanical activities [6,10]; the triboelectric nanogenerator usually requires a large relative displacement between two triboelectric parts [13]. By contrast, the piezoelectric generator is more desirable to harvest biomechanical energy for wearable power sources, especially for insoles or implantable applications where small displacements are required [20].

Human motion monitoring is of great importance in various areas such as rehabilitation, sports science, robotics, geriatric care and medical treatment. A lot of work has been done to monitor human motion. Pressure and force sensors [21,22,23], accelerometers [24,25], gyroscopes [26,27], goniometers [28] and ultrasonic sensors [29] are used alone or integrated together [30,31,32] to analyze human activities and gait, but these sensing systems all need external power sources which make them less compact. Without external power sources, biomechanical energy harvesters can serve as self-powered sensors to monitor human motion. Yi et al. proposed stretchable human motion sensors based on a triboelectric nanogenerator which can be applied to detect diaphragmatic breathing and joint movements [33], Yang et al. utilized a triboelectric nanogenerator for self-powered biomonitoring and human identification [34], Lee et al. reported self-powered sensor arrays based on triboelectric nanogenerators to map the distribution of foot pressure [35]. However, these self-powered sensors have vulnerable sensing abilities since their outputs generally depend on the initial surface triboelectric charges and are easily affected by environmental conditions such as humidity. In addition, these sensors are not capable of classifying human activities.

Here we report a portable, flexible piezoelectric energy harvesting insole which can classify human motions through the voltage waveforms generated by the piezoelectric effect. The insole is designed to be worn in a shoe without interfering with the gait. The piezoelectric staves are stretched when the foot presses on the insole. The characteristic of energy harvesting is verified both theoretically and experimentally. Such an insole has an average power over 100 μW, which enables it to be used as a direct power source for electronics like an electronic watch or a smart band. Information drawn from the waveforms of the voltage generated from different parts of the insole, proved to possess different features for different locomotion modes. This enables the insole to serve as a self-powered sensor for human motion recognition. Six kinds of human motion can be recognized, including normal walking, strolling, brisk walking, jogging, and ascending or descending stairs, through the characteristics of their voltage waveforms. We propose using peak numbers, time length and slopes of waveforms to judge the motion type. An accuracy of over 90% is achieved during the tests. Our work makes an attempt to extract human motion information from the piezoelectric energy harvester, expanding the practical applications of energy harvesters.

## 2. Design

**Figure 1:**
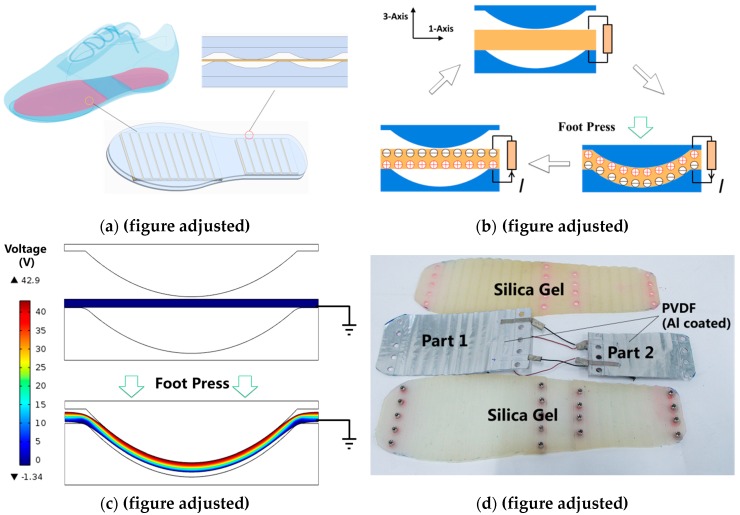
Design of a model of the self-powered insole. (**a**) Application and structure of the designed insole; (**b**) Working mechanism of the self-powered insole; (**c**) Numerical simulation results of the self-powered insole; (**d**) Photo of a fabricated insole.

The device was initially designed to be put directly into a shoe and to harvest the wasted energy generated by the foot sole during walking. Different parts of the insole reflect the distributions of pressure on them, so that the insole can be used to recognize human motion. The working principle is shown in Figure 1a,b. The structure of the device looks like a sandwich, as piezoelectric staves are inserted between the upper and lower plates on which wavy ribs and grooves are respectively arranged, as described in a previous work [20]. The piezoelectric staves, composed of multiple polyvinylidene fluoride (PVDF) films are fixed at both ends on the plates. When the upper plate is pressed, the PVDF films are stretched along the 1-axis. As a result, electrons are accumulated on the upper and lower surfaces of the films, and can be collected by means of electrodes. The upper plate goes up when the foot pressure decreases, then the PVDF films are released. The parasitic capacitors start to discharge at this moment, releasing reverse charge. The PVDF films return to zero potential when the release of reverse charge has ended.

Moreover, the voltage generated by the piezoelectric films is nearly in direct proportion to the force $F_{1}$ that stretches the stave. This proportion can be written as:

(1) $$U_{3}=\frac{h}{lw\varepsilon_{0}\varepsilon_{r}}d_{31}F_{1}$$

where $U_{3}$ is the voltage generated across the surfaces of PVDF that are perpendicular to the 3-axis, h ,w ,l are respectively the thickness (parallel to the 3-axis), width and length (parallel to the 1-axis) of PVDF film, $\varepsilon_{0}$ and $\varepsilon_{r}$ are the vacuum permittivity and relative permittivity of PVDF, $d_{31}$ is the piezoelectric constant of PVDF. Deformation of film thickness can be neglected, and elongation of length is designed to be less than 2% [20], which can also be neglected, so $U_{3}$ is nearly proportional to $F_{1}$. Thus $F_{1}$ is approximately proportional to the force applied by the foot on the upper plate $F_{foot}$, considering the mass of the upper plate is negligible:

(2) $$F_{1}\approx \frac{1}{2\sin\left(\alpha /2\right)}F_{foot}$$

where α is the central angle of the ribs and grooves. As a result, $U_{3}$ is nearly proportional to $F_{foot}$ which means the magnitude of voltage reflects the force applied on the upper plates. Feet exert force in different patterns people perform different movements so that there are various distributions of foot plantar pressure. It is possible to recognize the locomotion modes of people according to the variation of the plantar pressure distribution [36,37]. As the voltage generated by piezoelectric films is approximately in proportion to the force applied to the upper plates, our piezoelectric energy harvester is capable of recognizing different human movements through extracting relevant information of the foot plantar pressure distribution from the voltage waveform.

As shown in Figure 1c, the variation of electrical potential is numerically studied via COMSOL (a multi-physics simulation software), which verifies the working principle. The size of established model corresponds to one of the waves (12 mm in width along the 1-axis and 4 mm in height, the thickness of the PVDF films being 0.3 mm) that is arrayed on the plates. A potential difference of 42.9 V is generated across the piezoelectric stave when the upper plate is pressed down. In addition, a net positive charge accumulates on the upper surface of the stave. As shown in Figure 1d, both plates are made of injection-molded silica gel. PVDF films on which Al electrodes are coated are bonded together in parallel. Two pieces of multilayer PVDF films are fixed separately in the insole under the forefoot and heel of the foot. PVDF stacks are not placed under the arch of the foot as the arch hardly applies any pressure to an insole. According to the design mentioned above, the device finally fabricated has a shape similar to that of an insole for daily use, and it is thin enough (265 mm long, 100 mm wide, 10 mm thick) to be put into a normal shoe. Its mass is 239 g. The outline of the wavy ribs and grooves is an arch, the central angle of which is 27.7° and its chord length is 10 mm. In all, 10 waves and 6 waves are arrayed at equal intervals under the forefoot and heel, respectively, which are also defined as “part1” and “part2”. Both piezoelectric staves placed in front and behind are 8-layer stacks (with a size of 120 mm × 72 mm × 0.3 mm and 80 mm × 50 mm × 0.3 mm). The upper plate, PVDF stacks and lower plate are fixed together with bolts and nuts.

## 3. Characteristics and Applications for Directly Powering Electronics

### 3.1. Output Characteristics

**Figure 2:**
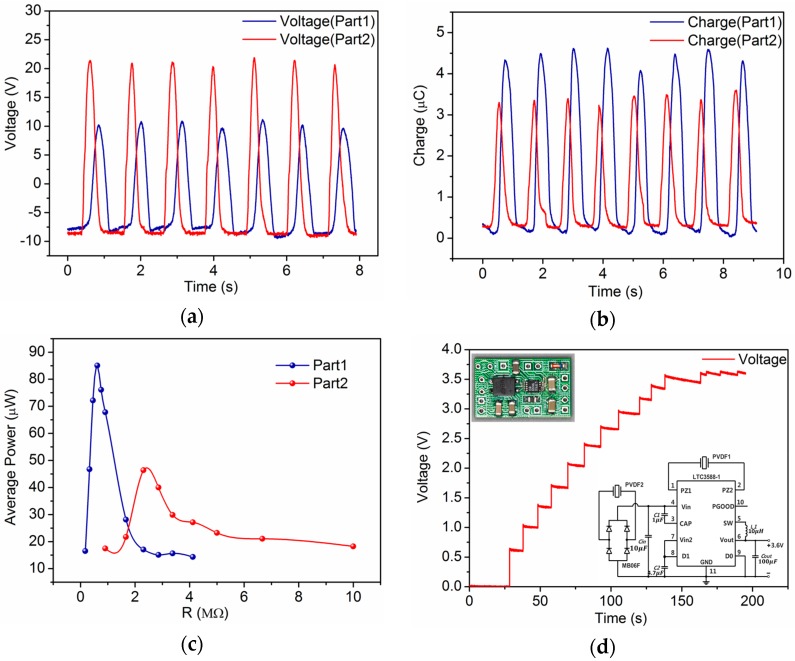

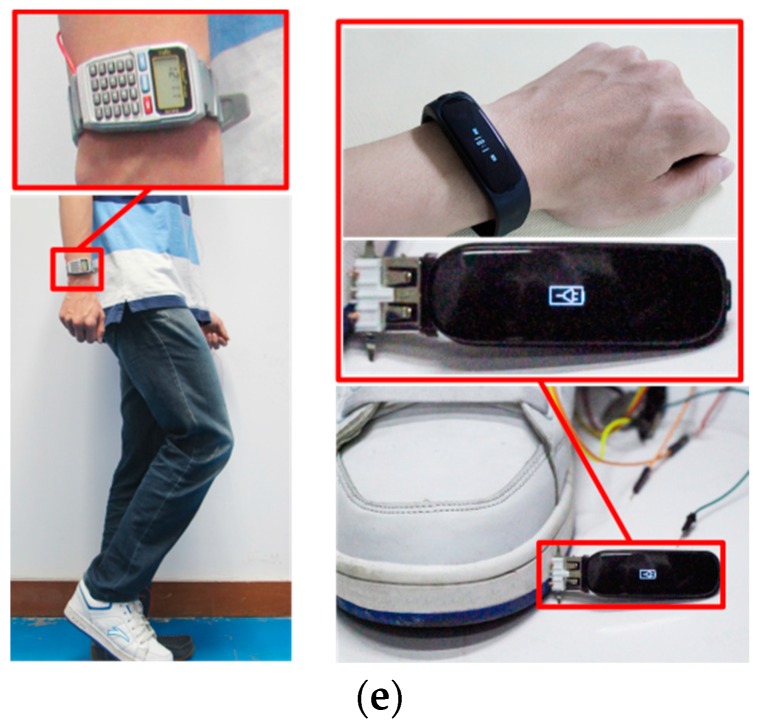
Typical electrical performance and application of the self-powered insole. (**a**) Open-circuit voltage output of the insole; (**b**) Short-circuit charge output of the insole; (**c**) Average power output from a load resistor; (**d**) DC ouput from the power management circuit; (**e**) Demonstration of a self-powered watch and a smart band being charged by the insole.

The voltage output and short-circuit transferred charge of the insole are measured when it is directly connected to an electrometer (mod. 6514, Keithley, Beaverton, OR, USA). The results are shown in Figure 2a,b. Normal human walking can drive part1 and part2 to generate output voltages of 20.8 V and 31.5 V, respectively, and a short-circuit transferred charge of about 4.6 μC and 3.5 μC while the device is embedded in a shoe as an insole. Figure 2c shows the output power while the insole is terminated with various load resistances. Average power is calculated by integrating the instantaneous power of the load. The electricity generated by PVDF films is AC power with voltage greater than 20 V and a current below 10 μA which cannot be used directly. A power-management circuit should be implemented to convert AC power to voltage-stable DC power so that the electric energy can be utilized.

As shown in Figure 2d the circuit is designed based on commercial nanopower energy harvesting chip-LTC3588-1 to ensure that the circuit is capable of harvesting energy from two piezoelectric energy sources (part1 and part2) [20]. The target voltage of LTC3588-1 is set as 3.6 V, which is suitable for most electronic applications. The output characteristic of the power management circuit is shown in Figure 2d when the insole is connected to it. The measurements show that the output voltage stabilizes at 3.45 V when the load of the circuit is 1 MΩ, which means the load power consumption is 11.9 μW and the transfer efficiency is 9.16%.

### 3.2. Applications to Commercial Systems

The self-powered insole is able to power commercial wearable devices through the power management circuit as shown in Figure 2e. The energy harvested from a man who wears a shoe (right side) with the insole stepping roughly at a frequency of 1 Hz, is capable of driving a commercial watch. The watch displays time on a LCD screen and it can also be used as a calculator. The insole can also serve as a power source to charge a smart band. The charge output is approximately 8 μC in every step which is measured as described above. Taking a smart band (such as a Mi-band) for instance, its battery is about 40 mAh and works 30 days on a single charge. If a person who wears the insole walks 15,000 steps every day, he will charge the band for 1 mAh in 30 days. Though the calculation is conducted theoretically, it is possible for the insole to prolong battery life.

## 4. Characteristics and Applications for Self-Powered Human Motion Recognition

The insole can also serve as a self-powered human motion detector which is able to judge daily movements such as normal walking, strolling, brisk walking, jogging, ascending stairs and descending stairs. Different human motion can be recognized by features extracted from various kinds of voltage waveforms, which reflect variations in plantar pressure.

### 4.1. Experiment Protocol

**Figure 3:**
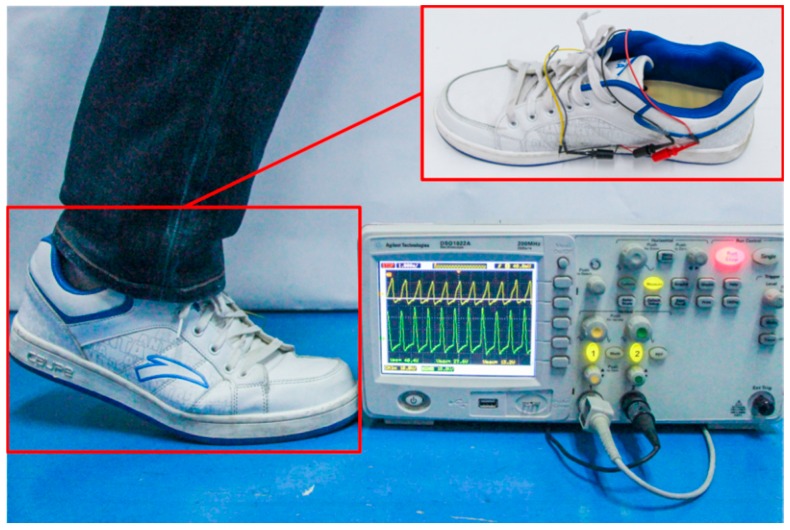
A photograph showing the insole in a shoe directly attached to an oscilloscope to record voltage waveforms generated under foot.

**Figure 4:**
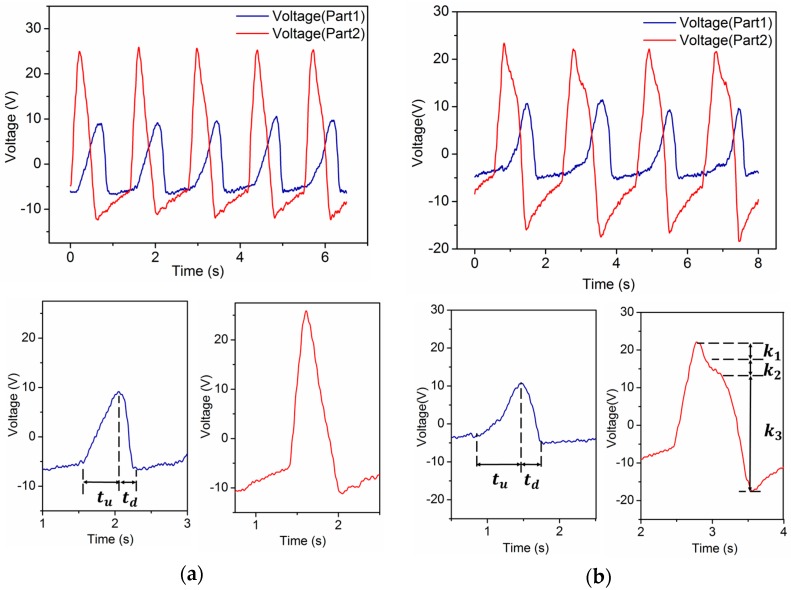
Waveforms and characteristics of voltage output from self-powered insole. (**a**) Waveforms and characteristics of normal walking, $t_{u}>t_{d}$ in voltage of part1; (**b**) Waveforms and characteristics of strolling, $t_{u}>t_{d}$ in voltage of part1, $\left|k_{2}\right|<\left|k_{1}\right|\approx \left|k_{3}\right|$ in voltage of part2.

**Figure 5:**
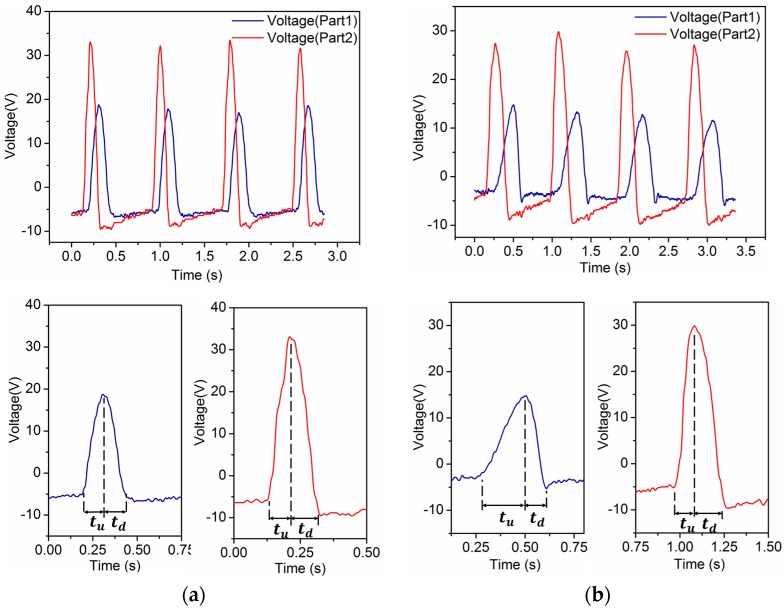
Waveforms and characteristics of voltage output from self-powered insole. (**a**) Waveforms and characteristics of jogging, $t_{u}\approx t_{d}$ in voltage of part1 and part2; (**b**) Waveforms and characteristics of brisk walking, $t_{u}-t_{d}$ in voltage of part1 is less than that of normal walking, while $t_{u}>t_{d}$.

**Figure 6:**
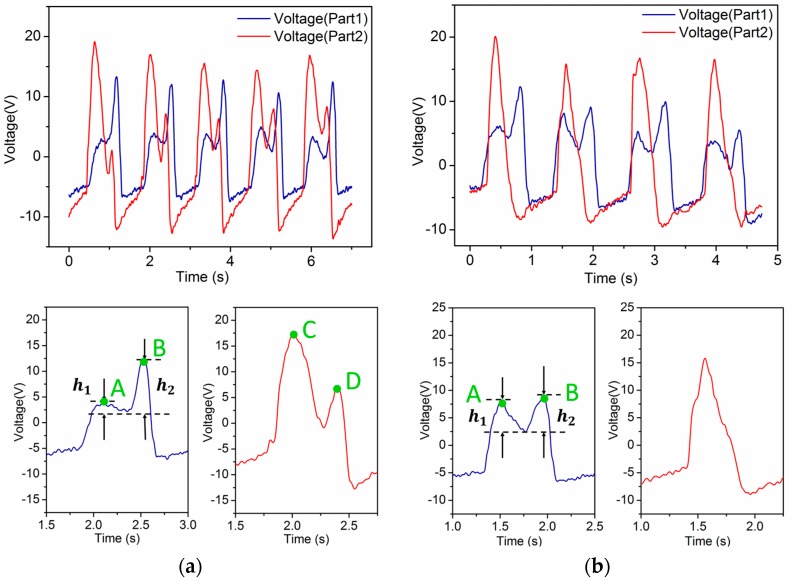
Waveforms and characteristics of voltage output from the self-powered insole. (**a**) Waveforms and characteristics of ascending stairs, double peaks in voltage of part1 and part2, and $h_{2}>h_{1}$; (**b**) Waveforms and characteristics of descending stairs, double peaks in voltage of part1, but not in part2.

Three healthy volunteers were invited to participate in an experiment to evaluate the motion recognition performance. The average height and mass of the volunteers are 177 ± 5.4 cm and 75 ± 7.9 kg (mean ± σ). Wearing a shoe embedded with the self-powered insole, they performed six locomotion modes: (a) normal walking; (b) strolling; (c) brisk walking; (d) jogging; (e) ascending stairs; and (f) descending stairs. Walking and jogging were conducted on flat marble ground. Volunteers normally walked and jogged in a straight line at their normal speed, and strolled and briskly walked at roughly 0.5–0.7 Hz and 1.5 Hz. Subjects were asked to ascend and descend stairs at 1 Hz. The steps of the stairs are 14.5 cm in height and 32 cm in width. As shown in Figure 3, two channels of data were collected directly from the insole by an oscilloscope. Each participant was invited to perform each mode of locomotion more than four times. Each activity lasted 10–15 s during the experiment. A total of 96 sets of data was collected, i.e., 16 sets for each locomotion mode. Three to four steps, or periods, are cut from each set of data for examination, so that some invalid waves are excluded, which were taken when participants were not ready. Typical waveforms of the six locomotion modes which are used as templates are shown in Figure 4, Figure 5 and Figure 6. They display the influence of foot plantar pressure distribution on the waveforms.

### 4.2. Analysis

During a normal walking period, the heel and forefoot merely strike the insole and then rise from it in turn. As a result, the waveforms of part1 and part2 generated by the process are sequential peaks. It is observed that the heel strikes rapidly but rises slowly, whereas the forefoot strikes slowly but rises fast. Accordingly, we define the period of time when the forefoot starts to contact the insole until the pressure reaches its maximum as the “pressure ascending time”, denoted as $t_{u}$, and the time from the pressure reaching its maximum to the forefoot leaving the insole as the “pressure descending time”, denoted as $t_{d}$, as is shown in Figure 4a. A typical feature of normal human walking is that $t_{u}>t_{d}$ for the forefoot.

While people are strolling, their heels and forefeet still strike and rise from the insoles in turn once during a period. But in contrast to walking, the shoes are pressed on the ground longer as strolling is much slower. As a consequence, the lengths of $t_{u}$ and $t_{d}$ are longer. However, a much more remarkable feature is that this extended contact time gives rise to a variation in the slopes of the waveforms during $t_{d}$, rather than going down at a constant slope, which is shown in Figure 4b as $\left|k_{2}\right|<\left|k_{1}\right|\approx \left|k_{3}\right|$ ($k_{i}$ is defined as slope). We can recognize strolling based on this feature.

The self-powered insole generates other kinds of waveforms when the wearer is walking briskly (without both feet leaving the ground at the same time) or jogging (both feet may leave the ground at the same time). The frequency of these two kinds of human motion is usually 1.5 Hz or higher. Their values of $t_{u}$ and $t_{d}$ for part1 and part2 are almost the same which means the ascending time and descending time with pressure on the insole are similar, as is shown in Figure 5a,b. This makes them different from other sorts of movements, but the forefoot’s total time of ascending pressure during brisk walking is a little longer than for jogging because both feet will not leave the ground at the same time. This distinguishes the one locomotion mode from the other.

As shown in Figure 6a,b, there are characteristics that differentiate ascending stairs and descending stairs from all other human motions. What makes them distinctive is that there are two peaks in one period in the waveforms generated by the forefoot during both ascending and descending stairs. The reason why they contain two peaks is that people have to keep their balance at first and then move to the next step whether they are going upstairs or downstairs. A difference still exists between these two locomotion modes. People put less pressure on the forefoot than on the heel to keep themselves steady when they initially step onto the next higher stair. When they are ready to move up to the next stair using the other foot, they mainly use the forefoot of the current foot to push. The forefoot is used not only to remain balanced but also to reduce speed to offset the kinetic energy generated by the descending center of gravity when people are initially step to a lower stair. Meanwhile, there is hardly any force applied by the heel striking. As the center of gravity of a person moves forward after the body is steady, the heel puts pressure on the insole, and again, the forefoot applies force next. This completes the whole movement. We can conclude from the above that there are double peaks in waveforms generated by both the forefoot and heel during ascending stairs. In this process, peak A is far lower than peak B, which is shown in Figure 6a as $h_{2}>h_{1}$. Peak C is obviously higher than peak D, but while descending stairs, peak A is approximately as high as peak B, if not higher. In addition, there is only one peak in the waveform generated by the heel during a period.

The algorithm is written using MATLAB. First of all, the algorithm detects the positions of wave peaks and valleys in each period of the waveforms with built-in function “findpeaks” and records their magnitudes as well. The heights of peaks and the distances between peaks are taken into account to exclude fake peaks. The values of $t_{u}$ and $t_{d}$ are calculated according to the positions of peaks and valleys. These characteristic parameters are extracted according to the templates, and are used to classify the rest of the data later. Secondly, the pattern recognition is conducted in sequence. If the waveforms generated under forefoot and heel are both double-humped in a single period, then we recognize the movement as “ascending stairs”. If the waveform of the forefoot is double-humped but that of the heel is not, then we recognize the movement as “descending stairs”. The movement can only be one of normal walking, brisk walking, strolling and jogging if it generates one peak in a period. The parameters $t_{u}$ and $t_{d}$ are used as criteria to classify these other modes. The feature of normal walking and strolling is that $t_{u}$ for the forefoot is obviously longer than $t_{d}$. If there is a sudden variation of slope during $t_{d}$ then, the movement is recognized as strolling. If not, then it is normal walking. To the waveforms which do not distinctly meet the conditions that $t_{u}>t_{d}$, the sum and difference of the values of $t_{u}$ and $t_{d}$ for the forefoot are used to distinguish the left two modes. If the sum is small and the difference is almost zero, the movement is recognized as jogging. If not, it is brisk walking.

### 4.3. Results

As mentioned above, 96 total sets of data were collected. In all, six sets were used as templates, 84 out of the other 90 sets of data can be correctly recognized. The results are shown in Table 1 by means of a confusion matrix. Each row of the table stands for which class the data is classified as in the algorithm. Each column stands for one mode of movement that the volunteers actually performed. Each value in the diagonal refers to the percentage of correctly classified movements. The values not on the diagonal represent the misclassified movements. In Table 1, normal walking is marked as a, strolling as b, brisk walking as c, jogging as d, ascending stairs as e and descending stairs as f. Interestingly, all of the data of volunteer 1 whose data was used as templates (18 sets left) can be accurately classified. A total of 66 sets of data for Volunteers 2 and 3 can be recognized correctly. The exceptions were that five sets of their strolling were recognized as normal walking and one set of their brisk walking was recognized as jogging. The total accuracy is 93.33%, self-detecting accuracy is 100%, and non-self-detecting accuracy is 91.67%.

## 5. Discussion

According to the experimental results, over 90% of the situations in which people perform normal walking, strolling, brisk walking, jogging, ascending and descending stairs can be successfully recognized utilizing the self-powered motion detector. It can be proved that the voltage waveforms generated by the self-powered insole contain features such as positions and magnitudes of peaks and valleys which can be extracted to recognize the locomotion modes of different people, for they exert force in a similar pattern when they are performing a certain kind of movement. Meanwhile, the gait of each person has tiny differences for which the self-detecting accuracy is 100%, much higher than the non-self-detecting value of 91.67%. It can be speculated that self-detecting is more effective.

Ascending and descending stairs, normal walking and jogging can be successfully recognized. Numerous misjudgments occurred in strolling, and the accuracy of strolling detection was merely 66.7%, which means strolling cannot be recognized effectively enough. We found that distinctions among different people during strolling are more obvious than during other motions as strolling is slower. This results in the previous criterion being inadequate for classification. The five sets of data that were falsely recognized also showed variations of slopes, but they were not as evident as the templates. This led to their being classified as normal walking. The reason why brisk walking was recognized as jogging is that the characteristics of the data quickly approaches a critical point between brisk walking and jogging. As shown in this case, human motion is not always stable which results in misjudgment. Therefore recognition should be based on several periods of data. The accuracy might be improved if more periods of stable waveforms are acquired. A lot of information that is contained in the waveforms is not taken into account in the current recognition scheme, such as relative magnitudes of peaks and valleys, intervals between adjacent peaks, etc. Additional research should involve analyzing the remaining information in the waveforms to improve the recognition accuracy. Future work shall also involve adding more regions to the insole to achieve more waveforms for voltage which contain information about gait. Also, only three volunteers participated in the experiments, which is not enough. More samples will be collected in future research.

The self-powered human motion detector can be used to monitor daily energy expenditure as it is wearable and easy to operate. Combined with other monitoring systems, it helps to improve the physical condition of a person. The average power harvested by the insole by stepping at 1 Hz is 0.13 mW which is higher than the consumption by most of the self-powered sensors and is capable of powering wearable devices to extend their battery life. In the future, the insole can be developed into an identity recognition device which discriminates among different people based on foot plantar pressure distributions while harvesting energy at the same time. It can also be utilized to analyze the gait of people as an adjuvant therapy of some diseases like Parkinson’s in future research.

## 6. Conclusions

We have developed a self-powered human motion recognition insole based on piezoelectric films. The insole converts foot pressure to stretching of PVDF films through a sandwich structure. It is flexible and thin enough to be worn in shoes. It has been demonstrated that the energy harvested during human walking could power wearable devices such as watches and smart bands. What’s more, the waveforms generated by the piezoelectric films also contain motion information which could be extracted to distinguish locomotion modes such as normal walking, strolling, brisk walking, jogging, and ascending or descending stairs. The characteristics of the waveforms are analyzed and an algorithm is written to recognize the various signals autonomously. In a test of three participants a total detection accuracy of 93.33% has been experimentally demonstrated.

## Figures and Tables

**Table 1:** Confusion matrix of experiment results.

Class	Actual Movements (% of Samples)					
	a	b	c	d	e	f
**a**	100	33.3	-	-	-	-
**b**	-	66.7	-	-	-	-
**c**	-	-	93.3	-	-	-
**d**	-	-	6.7	100	-	-
**e**	-	-	-	-	100	-
**f**	-	-	-	-	-	100
